# Anatomical Sketches and Diagrams, with Descriptions and References

**Published:** 1845-01

**Authors:** 


Anatomical Sketches and Diagrams, with Descriptions and References. By *Thomas Wormald* and *Andrew Melville M'Whinnie,* Demonstrators of Anatomy at St. Bartholomew's Hospital. 4to. Highley, 1844.

This work is now complete, and is in every way calculated to fulfil its object—that of presenting a series of clear and simple Views of the more important parts of the Body—furnishing a useful guide to the Student in the dissecting-room, and, from its character as a Book on Regional Anatomy, will be equally acceptable to the Surgeon. The subjects have been judiciously chosen, and the lithographic drawings, which are mostly coloured, are executed with great fidelity.

Amongst the most useful of the Plates, we might select those containing views of the Orbit and of the 5th and 7th Cerebral Nerves—those of the Neck in the order convenient for dissection—of this region the plans will be found very complete and instructive.

The views of the Chest and Abdomen are new and original—in connexion with the Vena Cava and Vena Portæ, we have illustrations of the Fœtal Circulation.

The Axilla, Bend of the Elbow-joint, and Groin—the Gluteal and Popliteal Regions are displayed, and recall to mind the relative anatomy of these parts.

We can most confidently recommend this Work, which, from its moderate expense, will be within reach of every Student.

